# Connection between self-esteem and discontinuation of medication

**DOI:** 10.1192/j.eurpsy.2024.1505

**Published:** 2024-08-27

**Authors:** S. Laabidi, O. Abidi, A. Aissa, R. Hosni, U. Ouali, R. Jomli

**Affiliations:** Psychiatry A, Razi hospital, MANOUBA, Tunisia

## Abstract

**Introduction:**

Self-esteem entails evaluating oneself positively and often involves the need to be special and above average without comparisons with others. It could play a role in many areas of the patient’s life.

**Objectives:**

The aim of the present study was to find the prevalence of self-esteem and investigate the associations between self-esteem and treatment adherence in patients with schizophrenia spectrum disorders.

**Methods:**

This study involved outpatients with schizophrenia spectrum disorders ,according to (DSM-V) diagnostic criteria, attending the Department of Psychiatry A, Razi hospital between august and September 30, 2023. The level of self-esteem was measured with Rosenberg’s Self-Esteem Rating scale (SERS) and treatment adherence with the Medical Adherence Rating Scale (MARS).Socio-demographic characteristics were also collected.

**Results:**

Thirty stabilized outpatients with schizophrenia (n=18), schizoaffective disorder (n=11), brief psychotic disorder (n=1) were included in the study. The mean (SD) age of the respondents was 43.2years; the mean number of Hospital admissions was 4.7.Almost two thirds of this population (63.33% ) had low self-esteem and 36.67 % had high self-esteem. The level of self-esteem did not differ between diagnostic categories. Self-esteem also positively correlated with higher education and negatively with an increased number of hospitalizations. However, no significant association was found between socio-demographic variables and self-esteem. Adherence was further negatively correlated with age and age of onset of disorders. Patients suffering from schizophrenia had the lowest adherence to treatment.The results of the present preliminary study suggest a positive correlation between the SERS total scores and the MARS scores. It was found that higher levels of self- esteem are related with higher levels of treatment adherence and lower levels of self- esteem are associated with discontinuation of medications without a psychiatrist’s recommendation. This connection was present in all diagnostic groups.

**Conclusions:**

This study shows positive relationship between self-esteem and treatment adherence. Further studies are needed to investigate whether self-esteem is a factor positively influencing adherence to treatment and show if self-esteem training programs like standard psychoeducation and cognitive behavioral therapy could be beneficial to improve treatment adherence among psychiatric patients.

**Disclosure of Interest:**

None Declared

